# Redox Mechanisms in Cisplatin Resistance of Cancer Cells: The Twofold Role of Gamma-Glutamyltransferase 1 (GGT1)

**DOI:** 10.3389/fonc.2022.920316

**Published:** 2022-05-20

**Authors:** Alfonso Pompella, Alessandro Corti, Athanase Visvikis

**Affiliations:** ^1^Dept. of Translational Research, University of Pisa Medical School, Pisa, Italy; ^2^UMR 7365 CNRS-Université de Lorraine, Vandœuvre-lès-Nancy, France

**Keywords:** cisplatin, drug resistance, gamma-glutamyltransferase (GGT), glutathione, biomarkers

## Abstract

Cisplatin (CDDP) is currently employed for the treatment of several solid tumors, but cellular heterogeneity and the onset of drug resistance dictate that suitable biomarkers of CDDP sensitivity are established. Studies on triple-negative breast cancer (TNBC) have recently confirmed the involvement of gamma-glutamyltransferase 1 (GGT1), whose enzyme activity expressed at the cell surface favors the cellular resupply of antioxidant glutathione (GSH) thus offering cancer cells protection against the prooxidant effects of CDDP. However, an additional well-established mechanism depends on GGT1-mediated matabolism of extracellular GSH. It was in fact shown that glycyl-cysteine – the dipeptide originated by GGT1-mediated GSH metabolism at the cell surface – can promptly form adducts with exogenous CDDP, thus hindering its access to the cell, interactions with DNA and overall cytotoxicity. Both mechanisms: mainainance of intracellular GSH levels *plus* extracellular CDDP detoxication are likely concurring to determine GGT1-dependent CDDP resistance.

The cytotoxic effects of cisplatin (CDDP) are in the first place mediated through its binding to DNA with the formation of intra-strand DNA adducts, leading to the inhibition of DNA synthesis and cell growth as well as to up-regulation of pro-apoptotic/down-regulation of anti-apoptotic genes/proteins, resulting in the induction of both intrinsic and extrinsic pathways of apoptosis. CDDP also induces production of reactive oxygen species (ROS), lipid peroxidation and activation of p53 signaling and other signal transduction pathways (1). Indeed, the molecular and (epi)genetic mechanisms accounting for the onset of CDDP resistance variably involve one or more of such cellular targets, as documented in several solid neoplasias including triple-negative breast cancer (TNBC) (2, 3).

Highly aggressivity, high recurrence rate and poor prognosis make the management of TNBC one of the hardest challenges in oncology. Treatment strategies over the last decades have been largely focused on chemotherapeutics such as taxanes and anthracyclines, but regimens based on CDDP – alone or in combination – have more recently taken the stage as a more versatile and promising tool against TNBC, especially in carriers of BRCA1 mutations or other deficiencies in the DNA repair systems making cells sensitive to clastogenic effects. Major difficulties are however represented by the frequent occurrence of CDDP resistance and the considerable heterogeneity of TNBC, leading to a wide variability in patients’ response to the drug. Several potential biomarkers are under investigation in order to identify patients most likely to benefit from CDDP, including homologous recombination repair deficiency, tumor infiltrating lymphocytes, TP53, cyclin-dependent kinase 2 expression, vascular endothelial growth factor and matrix metalloproteinase-9 (4–6).

An interesting study has recently described a novel cellular/molecular mechanism leading to CDDP resistance of TNBC, highlighting a crucial role for the expression of gamma-glutamyltransferase 1 (GGT1). An interplay of cancer cells with tumor associated macrophages (TAMs) was in fact described, in which TGF-β1 released by TAMs induces TNBC cells to secrete hepatic leukemia factor (HLF), which in turn transactivates GGT1 (7). The expression of this enzyme activity is shown to enable resistance to cisplatin by potentiating TNBC defenses against ferroptosis, the peculiar cell death mode consequent to iron-dependent lipid peroxidation. The finding is interpreted as the result of an improved cellular supply of the antioxidant tripeptide glutathione (GSH), enabling TNBC to protect themselves against the oxidant stress caused by CDDP and prevent the development of cytotoxic lipid peroxidation (7).

However, taking into account the complex set of effects produced by GGT1 enzyme activity (8), such interpretation is likely incomplete. The involvement of GGT1 in drug resistance of cancer cells has been repeatedly reported in quite a number of different neoplasias (reviewed in 9). GGT1 enzyme activity is located at the cell surface and catalyzes the cleavage of gamma-glutamic acid from GSH, thus effecting the first step in a salvage pathway of this critical cellular antioxidant from the extracellular space. In this way GGT1 can favor the maintainance of adequate intracellular GSH levels, and this has been long regarded as the sole mechanism underlying the protection offered by GGT1 against cytotoxicity of prooxidant drugs, including CDDP (10). In addition to its well-established genotoxic effects, the cytotoxicity of CDDP has been in fact variably attributed to production of reactive oxygen species following activation of NADPH oxidase (11) or mitochondrial dysfunction (12, 13). The programmed cell death process termed ferroptosis, in particular, is set in motion when cellular GSH levels are depleted to an extent such that the activity of glutathione peroxidase-4 (GPX4) is compromised. Once this has occurred, lipid peroxidation reactions are no longer regulated and can proceed up to the fragmentation of phospholipids with consequent derangement of cell membranes – a step catalyzed by the presence of free, redox-active iron. These processes are likely taking place in the experimental model used in the mentioned study (7), where the protection afforded by GGT1 expression in TNBC against ROS production, lipid peroxidation and cell death is indeed accompanied by increased levels of intracellular GSH.

A comprehensive interpretation of the role played by GGT1 in CDDP resistance cannot however ignore an additional mechanism documented by previous studies from our own laboratories. We could in fact demonstrate that glycyl-cysteine – the dipeptide originating from the cleavage of GSH effected by GGT1 at the cell surface – accumulates in the extracellular milieu of GGT1-transfected cells (**Table 1**) and is therefore liable to react with exogenous compounds such as CDDP. GSH itself reacts with CDDP in a 2:1 ratio, forming a complex consisting of a bi-dentate adduct in which both the sulfur and the amide nitrogen of the cysteine residues become bound to the drug (14). Such reaction is however remarkably slow, while formation of a similar adduct of CDDP with GSH metabolite glycyl-cysteine is approximately 10-fold quicker (15). Thus, due to the higher reactivity of its SH group as compared to that of parent tripeptide GSH, glycyl-cysteine can promptly form adducts with exogenous CDDP (**Figure 1C**) hence hindering it from accessing the cell, resulting in significantly decreased levels of DNA platination (**Figure 1B**). Overall, the occurrence of such reactions suggests that GSH metabolism effected by GGT1 expressed at the surface of cancer cells can actually mediate sort of an *extracellular detoxication* of CDDP, which adds to the antioxidant defense offered by intracellular GSH against CDDP-induced oxidative stress, and is likely a major factor concurring to prevent its cytotoxicity (**Figures 1A, D**) (14–16).

**Table 1:** Selective accumulation of the reactive GSH metabolite glycyl-cysteine in the extracellular space of GGT1-transfected melanoma c21/GGT cells.

µM	c21 cells	c21/GGT cells
GSH	4.69 ± 0.48	0.47 ± 0.11*
Glycyl-cysteine	0.15 ± 0.05	6.17 ± 0.19**

Low molecular weight thiols were assayed after 24 h cell culture in sulfur aminoacid-free (SAF) medium. Data are means ± SD of three detns. *p < 0.01; **p < 0.001. Modified, from Franzini et al. (13).

**Figure 1:**
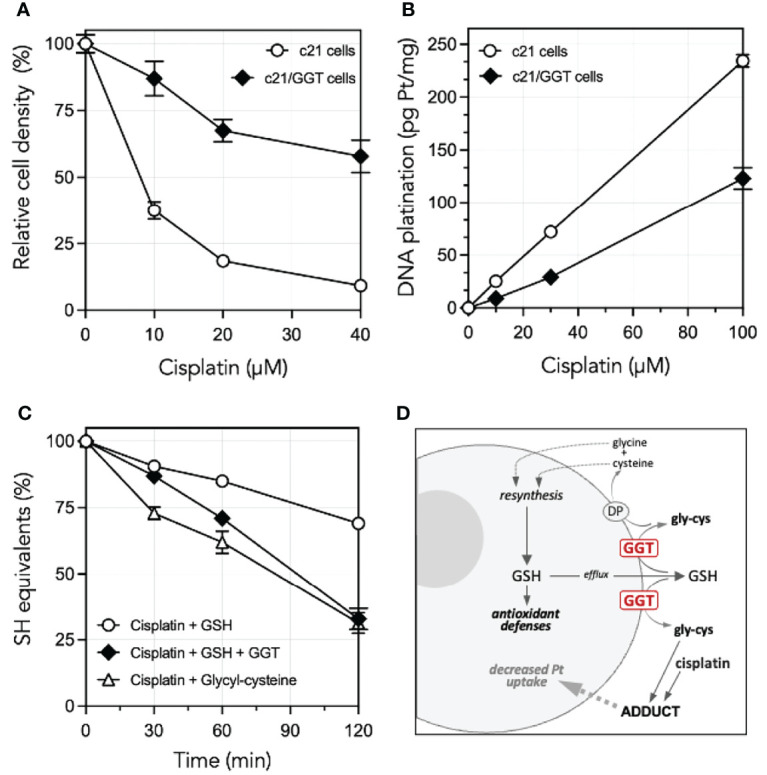
Cisplatin resistance of GGT1-expressing melanoma cells, degrees of DNA platination and differential reactivity of cisplatin with low mol. weight thiols. **(A)** Cisplatin sensitivity of c21 melanoma cells as compared to their GGT1-transfected (c21/GGT) counterparts. Cells were incubated 24 h with cisplatin and cell number was determined by WST-1 assay. Results are expressed as % of untreated controls, and are means ± SD (N = 12–36). **(B)** Corresponding levels of DNA platination in the same two clones as in A. Cells were exposed to cisplatin for 1 h. Platination was measured by inductively coupled plasma mass spectroscopy. Values shown are means ± SD (N = 3). **(C)** Reactivity with cisplatin with GSH in absence (〇) or presence of 200 mU/ml purified GGT1 (♦), or with the product of GGT1-mediated metabolism of GSH, glycyl-cysteine (Δ). SH groups were assessed at time intervals in a mixture containing 1.5 mM cisplatin and 3 mM GSH or glycyl-cysteine. Values shown are means ± SD (N = 3). **(D)** Proposed mechanisms underlying the GGT1-mediated resistance of cancer cells to cisplatin cytotoxicity. Modified, from Paolicchi et al. (14), Daubeuf et al. (15) and Franzini et al. (13).

In conclusion, the expression of GGT1 represents an important biomarker of CDDP resistance, and studies are warranted in order to investigate its ability to prevent ferroptosis in other malignant neoplasias besides TNBC. The development of CDDP resistance was observed to correlate with the activation of Keap1/Nrf2 transduction pathway in a series of solid tumors (6) and signalling through the Keap1/Nrf2 axis is indeed involved in GGT1 expression (17), raising the possibility that this may be the case in at least some patients. The finding of GGT1 expression should be anyway interpreted as a biomarker of CDDP resistance and an indication towards alternative treatments. In this latter respect, while precluding platinum-based treatments GGT1 expression may conversely offer unique therapeutic opportunities. The specific ability of GGT1 to cleave the gamma-glutamyl residue can in fact be exploited to activate glutathionylated pro-drugs, a pharmacologic strategy whose efficacy was documented in melanoma cells treated with the anti-angiogenic agent GSAO (18).

## Author Contributions

All authors conceived the article and discussed its content. AP wrote the main text and prepared the figures. All authors contributed to the article and approved the submitted version.

## Funding

The financial support by University of Pisa (Institutional funds 2021) is gratefully acknowledged.

## Conflict of Interest

The authors declare that the research was conducted in the absence of any commercial or financial relationships that could be construed as a potential conflict of interest.

## Publisher’s Note

All claims expressed in this article are solely those of the authors and do not necessarily represent those of their affiliated organizations, or those of the publisher, the editors and the reviewers. Any product that may be evaluated in this article, or claim that may be made by its manufacturer, is not guaranteed or endorsed by the publisher.

## Abbreviations

HLF, hepatic leukemia factor; GGT1, gamma-glutamyltransferase-1; Gly-cys, glycyl-cysteine; GSAO, 4-(N-(S-glutathionylacetyl)amino) phenylarsonous acid; GSH, glutathione; TAM, tumor associated macrophages; TNBC, triple-negative breast cancer.
